# Complex Probiotics Ameliorate Fecal Microbiota Transplantation-Induced IBS in Mice via Gut Microbiota and Metabolite Modulation

**DOI:** 10.3390/nu17050801

**Published:** 2025-02-26

**Authors:** Yuan Gao, Qinggele Borjihan, Weiqin Zhang, Lu Li, Dandan Wang, Lu Bai, Shiming Zhu, Yongfu Chen

**Affiliations:** 1 Key Laboratory of Dairy Biotechnology and Engineering, Ministry of Education, Inner Mongolia Agricultural University, Hohhot 010018, China; gy230218@126.com (Y.G.); qgl@mail.imu.edu.cn (Q.B.); spyzyll@163.com (L.L.); wdd18827555818@gmail.com (D.W.); bluu188@163.com (L.B.); zsmyxtk@126.com (S.Z.); 2 College of Life and Environment Science, Huangshan University, Huangshan 245041, China

**Keywords:** complex probiotic, irritable bowel syndrome (IBS), gut microbiota, metabolites, inflammation

## Abstract

**Background/Objectives:** Irritable bowel syndrome (IBS) is a highly prevalent functional gastrointestinal disorder. Emerging evidence implicates gut microbiota dysbiosis in IBS pathogenesis, and probiotic interventions targeting microbial modulation hold therapeutic promise. **Methods:** this study used fecal microbiota transplantation to establish a mouse model of IBS before evaluating the effects of the complex probiotic by using metagenomics and targeted metabolomics to explore the potential mechanism. **Results:** After 14 days, the probiotic relieved constipation, reduced inflammation and intestinal permeability, lowered 5-HT levels and increased serotonin transporter (SERT) expression in tissues. Metagenomic analysis showed a reduced inflammation-related species abundance. It also decreased fecal butyric acid, acetic acid and tryptophan levels in IBS mice. **Conclusions:** The probiotic complex effectively alleviated IBS symptoms in mice by modulating gut microbiota and fecal metabolites, providing insights for future IBS research and treatment.

## 1. Introduction

Irritable bowel syndrome (IBS), a common functional gastrointestinal disorder, is characterized by abdominal pain and abdominal distension, as well as changes in bowel habits or stool characteristics. However, it excludes organic diseases that can cause similar symptoms [1]. According to the Rome IV criteria, IBS can be classified into four subtypes, namely IBS with predominant constipation (IBS-C), IBS with predominant diarrhea (IBS-D), IBS with mixed bowel habits (IBS-M) and unclassified IBS (IBS-U), based on abnormal defecation characteristics, and these subtypes present with varying symptoms in clinical practice [2]. Recent studies suggest that the worldwide incidence of IBS is on the rise, especially in developing countries, where it ranges from 5% to 20% in the general population [3]. Substantial studies have demonstrated that the pathogenesis of IBS could be associated with changes in the brain–gut axis, dysregulation of gut microbiota and immunity, abnormalities in gastrointestinal motility and intestinal permeability, visceral hypersensitivity, as well as social and psychological factors [4,5]. Dysbiosis of gut microbiota is one of the main pathogenic mechanisms of IBS. Some studies have shown that the types and functions of gut microbiota in patients with IBS differ significantly from those in the healthy population, and these differences may be related to the generation and persistence of symptoms of IBS [6]. Imbalance of gut microbiota may lead to damage to the intestinal mucosal barrier, disturbance of the intestinal mucosal immune mechanism, intestinal metabolic disorders, and influence on the brain–gut axis, which ultimately leads to the occurrence, as well as deterioration, of IBS [7]. Although gut microbiota is associated with IBS, there are differences in the types of gut microbiota in patients with different IBS subtypes (e.g., diarrheal IBS-D and constipated IBS-C), which provides a rationale for individualized treatment of IBS, but the specific differences in the flora and how they affect the pathophysiology of IBS remain to be further investigated. In addition, it has been reported that a low fermentable oligosaccharides, disaccharides, monosaccharides and polyols (FODMAP) diet can alleviate symptoms, including abdominal pain and bloating, in IBS patients, while also improving their anxiety states and activity disorders [8]. However, such effects seem to be correlated with the patients’ genotypes [9]. Concurrently, the low FODMAP diet, which modifies eating habits and restricts certain dietary components, can profoundly affect the composition and metabolism of gut microbiota, potentially leading to negative impacts on long-term health [10]. Similarly, pharmacological treatments for IBS, such as rifaximin, loperamide, colesevelam hydrochloride, selective serotonin reuptake inhibitors and antidepressants, are often accompanied by side effects such as nausea, hypotension and sexual dysfunction, just to name a few [11,12]. Therefore, current dietary and pharmacologic therapies are not ideal, as they are only effective in some IBS patients while often having notable side effects. Therefore, further research and development of new therapeutic approaches for IBS are essential.

In 2001, the Food and Agriculture Organization (FAO) and the World Health Organization (WHO) officially defined probiotics as “live microorganisms that, when administered in adequate quantities, provide a host with health benefits” [13]. Probiotics inhibit the adhesion of pathogens to intestinal epithelial cells by competitively regulating the production of bacteriocins, short-chain fatty acids and biosurfactants by the gut microbiota. Furthermore, they can enhance intestinal barrier function by down-regulating mild mucosal immune response, enhancing the mucus barrier, and stimulating the synthesis of tight junction proteins, while improving intestinal immunity by inhibiting pro-inflammatory cytokines [14,15]. So far, research has shown that IBS patients exhibit reduced gut microbiota diversity, especially in terms of decreased Firmicutes and increased Bacteroidetes compared with healthy individuals [16]. In contrast, other studies have reported a higher Firmicutes-to-Bacteroidetes ratio in IBS patients [17]. So far, the reason behind such inconsistent results remains unclear. However, Sun et al. [18] found decreased *Clostridium* levels in the feces of IBS patients experiencing reduced symptoms after treatment with *Clostridium butyricum*, possibly due to the down-regulation of fatty acid metabolism, β-alanine metabolism, tryptophan metabolism and other signaling pathways. Similarly, Niu et al. [19] observed that supplementation with multiple probiotic strains was more effective at improving IBS symptoms, including symptom persistence, abdominal pain and bloating compared with a single-strain probiotic. Finally, Preston et al. [20] found that a combination of *Lactobacillus acidophilus CL1285*, *Lactobacillus casei LBC80R* and *Lactobacillus rhamnosus CLR* significantly alleviated various clinical symptoms (except abdominal pain) in IBS patients and effectively enhanced their quality of life.

Complex probiotics is a novel probiotic powder formulation consisting of *Lacticaseibacillus paracasei Zhang* (LCZ), *Bifidobacterium animalis subsp. lactis V9* (V9) and *Lactiplantibacillus plantarum P-8* (P8). LCZ prevents endotoxin- and d-galactosamine-induced liver injury in rats by enhancing antioxidant and anti-inflammatory capacity and reduces pro-inflammatory cytokine production and hepatic inflammation [21]. Numerous animal studies have shown that supplementation with *Lactobacillus plantarum P-8* improves the body’s immune response and antioxidant capacity and has an inhibitory effect on intestinal pathogens [22]. It has been suggested that fermented milk containing *Bifidobacterium lactis V9* may alleviate symptoms of constipation by altering the intestinal microbiota [23]. Previous studies have shown that the complex of these three probiotics is an excellent probiotic product, which then increases the number of *Bifidobacteria*, *Lactobacillus* and other probiotics in the intestinal tract while decreasing the levels of harmful bacteria. In addition, these three bacteria balance the intestinal microbiota and have some anti-inflammatory effects, thereby reducing abdominal discomfort in patients with irritable bowel syndrome. Finally, clinical studies have shown that these three probiotic complexes can improve IBS-related symptoms [24,25,26].

Therefore, in this study, an IBS model was constructed through the transplantation of fecal bacteria before assessing the number of 2-h fecal pellets, fecal water content, gastrointestinal transit time, small intestinal propulsion rate, levels of inflammatory factors, histopathological changes, the 5-HT and serotonin transporter (SERT) content in colon and brain tissues, as well as the gut microbiota composition and metabolites. Furthermore, the effects of the complex probiotic on IBS were explored alongside its potential mechanisms. This research provides a scientific foundation for the innovative application of the complex probiotic in the prevention and treatment of IBS, with the results providing strong support for future applications in clinical research.

## 2. Materials and Methods

### 2.1. IBS Animal Experimental Design

Thirty 7-week-old GF-grade male C57BL/6J mice were purchased from Shenzhen Jintuo Bio-technology Co., Ltd. (Shenzhen, China; Laboratory Animal Production License No. SCXK (Guangdong) 2023-0065). The experimental animals were housed in a GF-grade animal house with an ambient temperature of 22 ± 2 °C, a humidity of 45% ± 10% and a 12-h light/dark cycle. Throughout the experiments, the mice were kept in isolation bags with free access to food and water. All animal experiments were conducted in accordance with the guidelines established by the Laboratory Animal Protection and Ethics Committee of Shenzhen Jintuo Biotechnology Co. (number: JTAW20230225-1).

Preparation of fecal suspension: 1. On the day of fecal microbiota transplantation, thaw frozen fecal samples (healthy human feces were provided by three volunteers from the Affiliated Hospital of Inner Mongolia Medical University, while feces from IBS-D patients were provided by three patients with severe IBS-D from the same hospital, see S1 for specific information.) in a thermostatic water bath at 37.5 °C for 10 min. 2. Open a clean bench and sterilize with ultraviolet light for 30 min. 3. Place autoclaved centrifuge tubes, gauze, filters, beakers, etc. on a clean bench. 4. Resuspend fresh or pre-frozen stool samples in 5% (*w*/*v*) sterile phosphate-buffered saline (PBS) or sterile sodium chloride solution (0.9%), dilute 200 mg of stool to a volume of 2 mL and vortex the stool until no large particles are visible. 5. Stir the feces until no large particles are visible; remove large particles from the feces by filtration through a 200 mesh sterile sieve, followed by filtration through 400 and 800 mesh sterile sieves to remove undigested food and smaller particles. 6. Collect the filtrate obtained through an 800 or 1000 mesh sieve in a sterile centrifuge tube. 7. Vortex the resuspension for 5 min. The resuspension was centrifuged at 600× *g* for 5 min at 4 °C using a centrifuge (Thermo Fisher Scientific, Waltham, MA, USA) to remove insoluble material. 8. Immediately transfer the fecal suspension obtained by centrifugation to an isolator according to the requirements for aseptic transfer of mouse fecal transplants. Dispense and freeze unused fecal suspensions on a clean bench.

**Figure 1:**
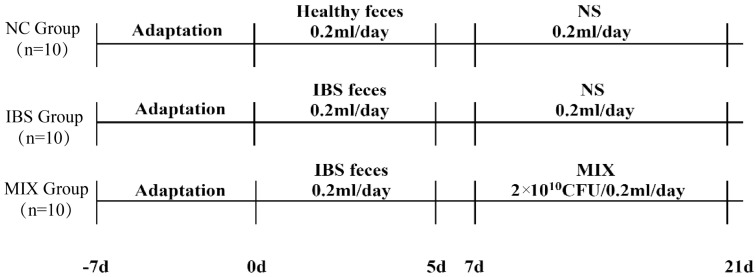
Experimental design.

One week after acclimatization of the experimental animals, mice were randomly assigned to one of the following three groups (Figure 1) using Simple Grouping J software ver. 1.0.5 (H&T, Osaka, Japan): the NC group, the IBS group, or the MIX group (10 mice per group) [27,28]. Mice in the NC group received 0.2 mL of healthy human fecal microbiota, while those in the IBS and MIX groups were gavaged with 0.2 mL of fecal microbiota from IBS patients for five days, followed by a two-day break. As of the 7th day, mice in the MIX group were given 0.2 mL of a mixed bacterial solution (2 × 10^10^ CFU/day of live bacteria) daily, while those in the remaining two groups were gavaged with 0.2 mL of saline.

Samples of feces and serum (centrifuged for 10 min at 3000 rpm and 4 °C) as well as brain tissues and colon tissues were collected from the mice on days 0, 7 and 21 (No animals were excluded from the analysis in this experiment due to poor health or experimental manipulation errors. All experimental animals and data points were recorded and used in the evaluation of results and statistical analysis. Outcome assessment was performed by researchers who were not involved in the experimental manipulation to ensure the blinding of the assessment process and to avoid bias.). All samples were immediately frozen at −80 °C and tested within one week.

*Lacticaseibacillus paracasei Zhang*: It comes from the grassland of Xilingol, Inner Mongolia, and is one strain of probiotic bacteria screened from 243 strains of lactobacilli isolated from 43 samples of traditional fermented sour horse milk; *Bifidobacterium animalis subsp. lactis V9*: A strain of acid-resistant probiotic selected from 31 strains of Bifidobacterium bifidum isolated from the feces of 12 healthy Mongolian children. *Lactiplantibacillus plantarum P-8*: It is an excellent probiotic strain isolated and screened from naturally fermented sour cow’s milk samples of herdsmen’s families on the grassland of Ulatzhong Banner, Bayannur City, Inner Mongolia. The complex probiotic used in this study was a mixture of *Lacticaseibacillus paracasei Zhang*, (3 × 10^9^ CFU/g), *Bifidobacterium animalis subsp. lactis V9*, (4 × 10^9^ CFU/g) and *Lactiplantibacillus plantarum P-8*, (3 × 10^9^ CFU/g), which were combined at a viable bacterial count ratio of 3:4:3. These three strains were provided by the *Lactobacillus* strains resource bank of Inner Mongolia Agricultural University.

### 2.2. Number of Fecal Pellets and Water Content

Defecation parameters, including fecal weight, dry fecal weight, fecal water content and the count of fecal pellets, were assessed for the mice. For this purpose, the mice were individually confined to separate cages and observed for a period of two hours. During this time, the number of fecal pellets and the wet weight of the feces were recorded. The feces were then dried to a constant weight at 37 °C to obtain their dry weight. From the readings, the final water content of the feces was calculated according to the following formula: (wet weight − dry weight)/wet weight × 100%.

### 2.3. Intestinal Permeability

An in vivo intestinal permeability assay was performed using FITC (Fluorescein isothiocyanate)-labeled dextran (Sigma-Aldrich, St. Louis, MO, USA). Briefly, the mice were fasted for 4 h without access to water, after which a FITC-dextran solution (50 mg/mL) was administered by gavage at a volume of 10 μL/g (followed by normal diet and water intake). After 4 h, the animals were anesthetized, and blood samples (80–100 μL per mouse) were collected via orbital sampling (about four drops). These blood samples were protected from light until serum separation. The serum was diluted in a ratio of 1:1 with PBS and added to a 96-well plate. FITC concentration was then measured at an excitation wavelength of 485 nm and an emission wavelength of 528 nm (Molecular Devices, San Jose, CA, USA), with serially diluted FITC used as a standard. Intestinal permeability was eventually assessed by measuring the amount of FITC-dextran that crossed the intestinal epithelial barrier into the bloodstream after oral administration.

### 2.4. Gastrointestinal Transit Time

Gastrointestinal transit time (GITT) was measured in mice using a carmine solution. For this purpose, the mice were deprived of food for a period of 16 h prior to the commencement of the experiment and were placed in individual cages. Each animal was then gavaged with 0.2 mL of 0.5% methylcellulose containing 6% carmine. The interval between oral administration and the appearance of the initial red fecal pellet was documented as the GITT (on day 8). Furthermore, to assess small intestinal propulsion rates, these mice were similarly gavaged with 0.3 mL of oral gavage of a 0.5% methylcellulose solution containing 6% carmine red. Half an hour later, the mice were euthanized, and the intestines from the pylorus to the cecum were isolated. The rate of propulsion in the small intestine was calculated as a percentage of the total distance traversed by the carmine red solution relative to the length of the small intestine.

### 2.5. Histological Analysis

Colon samples were collected from mice as previously described [29]. The terminal section of the colon (2 cm proximal to the anus) was rinsed with phosphate-buffered saline (PBS) and promptly preserved in a 4% paraformaldehyde solution following dissection. The fixed colon tissues were then dehydrated using an ethanol gradient, and after being embedded in paraffin, they were cut into 5-micrometer-thickness. The samples were finally stained with hematoxylin and eosin (H&E) and then examined in detail using an optical microscope from Shanghai Si Chang Yao Optical Instrument Co. (Shanghai, China). Specific scoring criteria and descriptions were added to the schedule (Table S2).

### 2.6. ELISA

Serum samples: Serum was obtained by centrifugation of the collected blood samples for 15 min at 3000× *g* and 4 °C. Tissue samples: After cutting the specimens, 1 g of tissue was weighed, 9 mL of PBS at pH 7.2–7.4 was added and the specimens were homogenize adequately using a homogenizer. The samples were centrifuged for about 20 min (2000–3000 rpm) and the supernatant was carefully collected. The cytokine levels in the serum samples (IL-6: MM-0163M2, TNF-α: MM-0132M2, IL-1β: MM-0040M2, IL-10: MM-0176M2), as well as the levels of 5-HT (MM-0443M2) and SERT (MM-47975M2), in colon and brain tissue samples were then quantified using appropriate ELISA kits (Jiangsu Meimian Industrial Co., Ltd., Yancheng, China) according to the manufacturer’s protocols [30,31,32].

### 2.7. Fecal DNA Extraction, Sequencing and Analysis

Fecal samples were processed to recover DNA using the QIAamp Stool Mini Kit (Beijing Noblelight Technology Co., Beijing, China), following the producer’s recommended protocol. Subsequently, the integrity and purity of DNA were determined by employing 1% agarose gel electrophoresis and a NanoDrop spectrophotometer. Afterward, the concentration of DNA was assayed using a Qubit^®^ 2.0 Fluorometer (Thermo Fisher Scientific, Waltham, MA, USA). In this case, samples with an OD value between 1.8 and 2.0 and a DNA content above 1 μg were deemed suitable for library construction.

For library preparation, 1 μg of genomic DNA from each sample was fragmented into pieces of approximately 350 bp using a Covaris (Covaris, Woburn, CT, USA) Ultrasonic Crusher. This was followed by a series of steps, including end repair, addition of A-tails, adapter ligation, purification and PCR amplification, to generate the final library. After the preparation process, preliminary quantification was performed with a Qubit 2.0 (Thermo Fisher Scientific, Waltham, MA, USA), and the library was diluted to 2 ng/μL. The fragment sizes were then measured with an Agilent (Agilent, Santa Clara, CA, USA) 2100 bioanalyzer, and upon confirming that the fragment size complied with the required criteria, the library’s effective concentration was ascertained via qPCR (the effective concentration of the library exceeded 3 nM) to verify its integrity. After the quality assessment, various libraries were combined according to their effective concentrations and intended data yield before sequencing on an Illumina PE150 platform. Each sample generated approximately 3 GB of downstream data. The resulting metagenomic data were then subjected to quality control, including de-hosting, to remove low-quality and contaminating sequences before using HUMAnN2 for taxonomic annotations of the sequences.

### 2.8. Determination of Fecal Short-Chain Fatty Acids and Tryptophan

After 0.1 g of fecal sample was weighed into a 2 mL homogenizing tube containing 1.0 mm zirconia beads, 1 mL of 50% methanol in water was added. The mixture was then homogenized at 3 m/s for 30 s with a homogenizer (Omni International, Kennesaw, GA, USA), and the process was repeated twice. The samples were then centrifuged at 4 °C 12,000× *g* for 10 min, with 40 μL of the resulting supernatant subsequently combined with 20 μL of 200 mM 3-NPH (3-nitrophenylhydrazine) and 20 μL of 120 mM EDC (*N*-(3-dimethylaminopropyl)-*N*’-ethylcarbodiimide hydrochloride)-6% pyridine. The samples were then shaken and centrifuged prior to a 30-min incubation at 40 °C. Following incubation, 1920 μL of 50% methanol in water was added, and after shaking and centrifugation, the supernatant was collected and filtered through a 0.22 μm microporous membrane filter (Sigma Aldrich, Saint Louis, MO, USA). The filtered solution was then transferred to the upper sample bottle for further analysis. Targeted metabolomics of fecal samples was performed using LC-MS. The conditions for the detection of the analytes were optimized based on the protocol established by Li et al. [33]. A chromatographic column (2.1 × 100 mm Phenomenex Kinetex^®^ 2.6 μm EVO C18 100A (Phenomenex, Torrance, CA, USA)) was employed with the following detection parameters: a column temperature of 40 °C, a flow rate of 0.4 mL/min and an injection volume of 2 µL/sample. Mobile phases A and B were composed of 5% methanol in water with 0.1% formic acid and pure methanol with 0.1% formic acid, respectively. The specific elution gradient procedures are also shown in Table S3. Subsequently, mass spectrometry scanning was conducted utilizing an electrospray ionization (ESI) source in conjunction with multiple reaction monitoring (MRM) mode. The nebulizing gas and the auxiliary heating gas were configured at 50 psi, whereas the air curtain gas was adjusted to 35 psi. The temperature of the desolvent gas and the ionization voltage were set at 550 °C and 4500 V, respectively. The acquired data were quantitatively evaluated using the SCEIX OS 3.4 software of the UPLC-MS/MS 6500+ system (AB Sciex Pte. Ltd., Boston, MA, USA).

### 2.9. Statistical Analysis

All results are expressed as mean ± standard error of the mean (SEM). The statistical significance of the observed differences between groups was then assessed using the nonparametric Mann–Whitney rank sum test, with significant differences indicated by *p*-values < 0.05. The resulting data were subsequently plotted using Origin 2021. For structural analysis of the fecal microbial communities, principal coordinate plots were generated based on Bray–Curtis distances, and correlation heatmaps were constructed using the OmicStudio tool (https://www.omicstudio.cn accessed on 5 April 2024).

## 3. Results

### 3.1. Effects of Complex Probiotic on the Pathological Indicators of IBS

**Figure 2:**
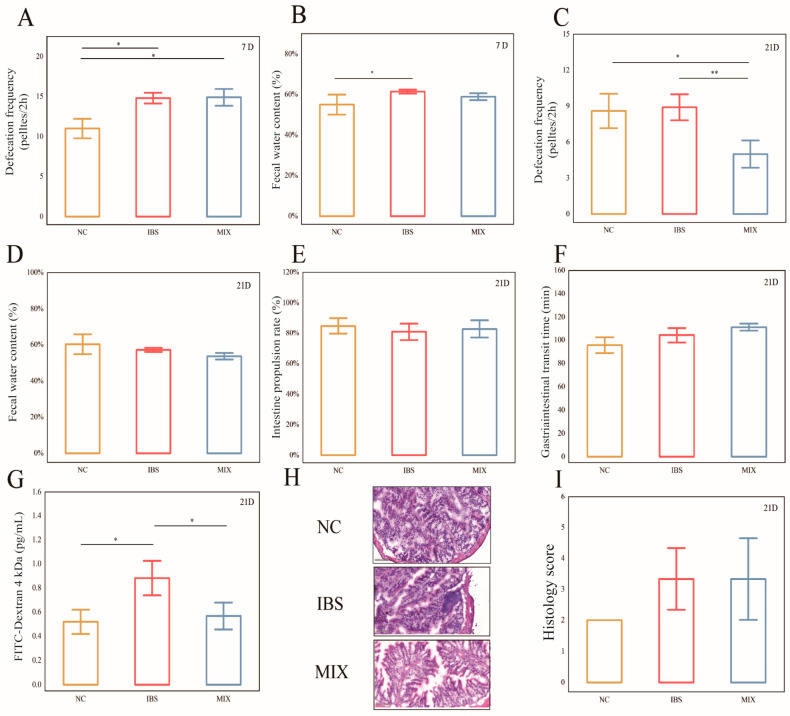
Treatment with a complex probiotic reduced disease severity in IBS mice (*n* = 10). (**A**) Number of 2-h fecal pellets on day 7. (**B**) Fecal water content on day 7. (**C**) Number of 2-h fecal pellets on day 21. (**D**) Fecal water content on day 21. (**E**) Small bowel propulsion rate. (**F**) Gastrointestinal passage time. (**G**) FITC (Fluorescein isothiocyanate)-labeled dextran content. (**H**) Representative micrographs of HE-stained sections of colon tissues (20× magnification). (**I**) Histological scores. Data are expressed as mean ± SEM. Mann–Whitney rank sum test. Notable disparities are signified by * (*p* < 0.05), ** (*p* < 0.01).

After modeling, mice in the IBS and MIX groups exhibited significantly higher 2-h fecal pellet counts relative to those in the NC group (Figure 2A: NC and IBS, *p* = 0.015; NC and MIX, *p* = 0.043), with the fecal water content also being markedly higher in the IBS group compared with the NC group (Figure 2B, *p* = 0.033). However, by day 21, the 2-h fecal pellet count in the MIX group was notably reduced compared with the IBS group (Figure 2C, *p* = 0.004), thus indicating that the complex probiotic alleviated diarrhea in IBS mice. Furthermore, at this point, the three groups were not significantly different in terms of their fecal water content, small intestinal propulsion rate or gastrointestinal transit time (Figure 2D–F). Additionally, relative to the NC group, the FITC concentration of the IBS group was markedly higher (*p* = 0.035), but it was significantly reduced after the complex probiotic intervention (Figure 2G, *p* = 0.046). HE-stained sections of colonic tissues from the three groups of mice were examined and scored under a light microscope (Figure 2H,I), with the results revealing no significant differences in the colonic tissue scores of the three groups.

### 3.2. Effects of Complex Probiotic on the Inflammatory Factors in IBS

**Figure 3:**
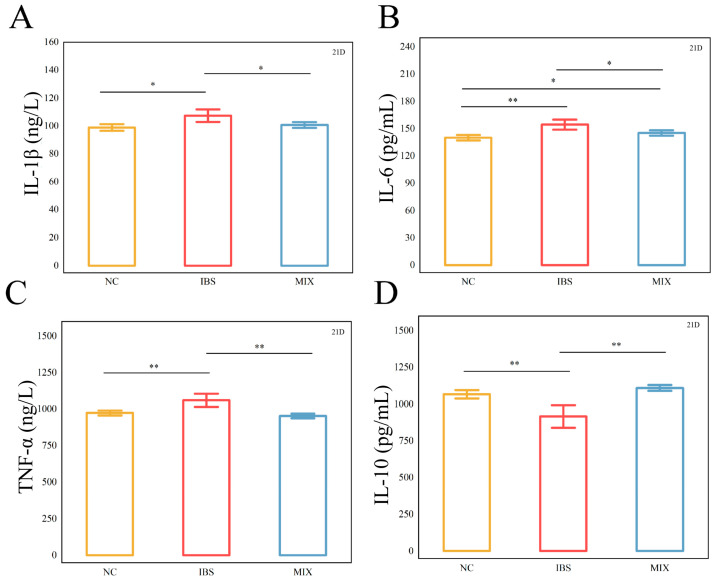
Treatment with a complex probiotic modulated the immune factors in IBS mice (*n* = 10). (**A**) Serum levels of IL-1β. (**B**) Serum levels of IL-6. (**C**) Serum levels of TNF-α. (**D**) Serum levels of IL-10. Data were expressed as mean ± SEM. Mann–Whitney rank sum test. Notable disparities are signified by * (*p* < 0.05), ** (*p* < 0.01).

The dysregulated expression of inflammatory factors can exacerbate the progression of IBS. The outcomes of the inflammatory cytokine measurements are depicted in Figure 3. The concentrations of serum pro-inflammatory cytokines IL-1β, IL-6, and TNF-α were markedly higher in the IBS group relative to the NC group (*p* < 0.05, *p* < 0.01, *p* < 0.01), while that of the serum anti-inflammatory cytokine IL-10 was markedly lower (*p* < 0.01). After the complex probiotic intervention, these trends were reversed in the MIX group relative to the IBS group. However, even though the complex probiotic reduced IL-6 levels, they remained markedly different from those in the NC group (*p* < 0.05). Altogether, these results imply that a complex probiotic can effectively reduce serum inflammatory factor levels.

### 3.3. Effects of Complex Probiotic on IBS 5-Hydroxytryptamine and Serotonin Transporter

**Figure 4:**
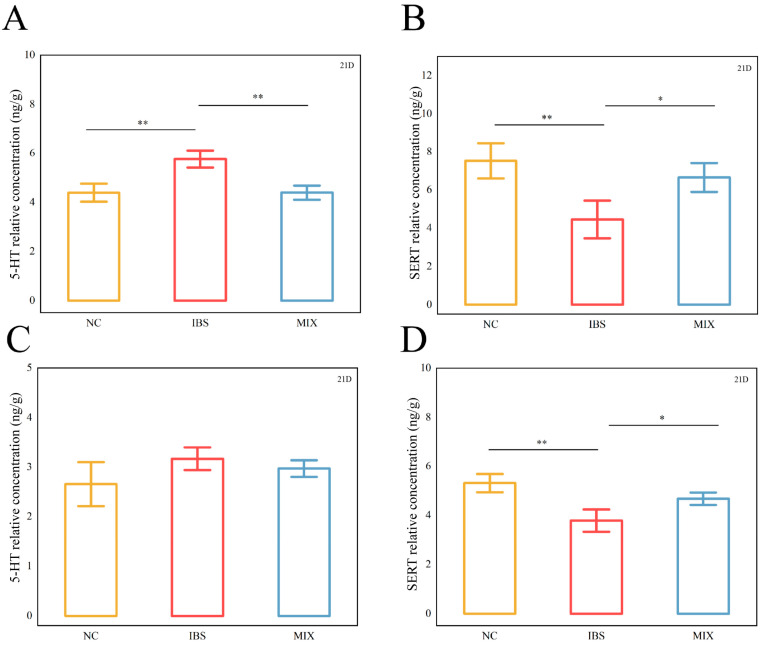
Treatment with a complex probiotic modulated 5-HT (5-hydroxytryptamine) and SERT (serotonin transporter) in IBS mice (*n* = 10). (**A**) 5-HT content in colon tissues. (**B**) SERT content in colon tissues. (**C**) 5-HT content in brain tissues. (**D**) SERT content in brain tissues. Data are expressed as mean ± SEM. Mann–Whitney rank sum test. Notable disparities are signified by * (*p* < 0.05), ** (*p* < 0.01).

5-HT plays a pivotal role in intestinal motility, and functional changes in SERT can influence 5-HT activity, which, in turn, may affect the pathogenesis of IBS. The ELISA results (Figure 4) showed that, in the colonic tissues, 5-HT levels were markedly elevated in the IBS group, while SERT levels were significantly lower compared with those in the NC group (Figure 4A,B, *p* = 0.006, *p* = 0.007). After intervention with a complex probiotic, the MIX group displayed markedly lower 5-HT levels but markedly elevated SERT levels relative to the IBS group (Figure 4A,B, *p* = 0.006), with the values approaching those of the NC group. Furthermore, in the case of the brain tissues, mice in the IBS group had significantly lower SERT levels compared with those of the NC group, while their 5-HT levels, despite seeming higher, were not significantly different (Figure 4C,D, *p* = 0.003). Following the complex probiotic intervention, the MIX group showed a trend toward lower 5-HT levels compared with the IBS group, although this difference was not significant, while SERT levels were significantly higher (Figure 4C,D, *p* = 0.030).

### 3.4. Effects of Complex Probiotic on Gut Microbiota in IBS

**Figure 5:**
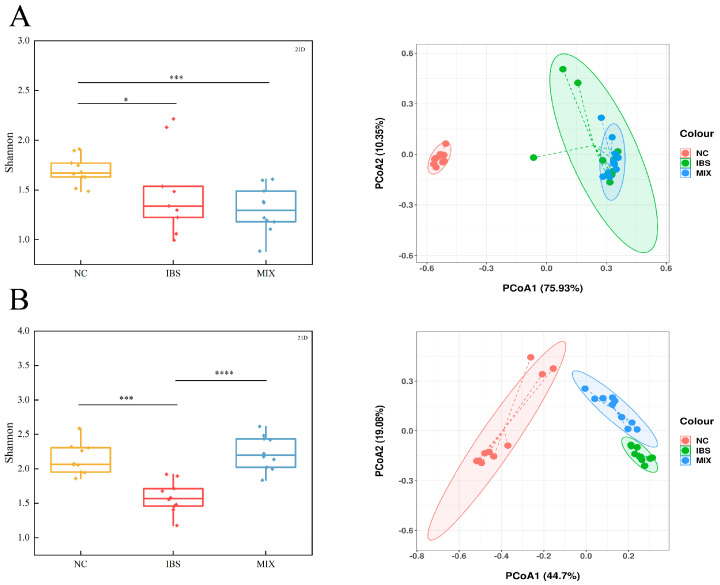
Treatment with a complex probiotic improved variety of the gut microbiome in IBS mice (*n* = 10). (**A**) Shannon index and principal coordinate analysis (PCoA, Bray–Curtis distance) at day 7. (**B**) Shannon index and principal coordinate analysis (PCoA, Bray–Curtis distance) at day 21. Mann–Whitney rank sum test followed by ANOSIM test. Notable disparities are signified by * (*p* < 0.05), *** (*p* < 0.001) and **** (*p* < 0.0001).

Dysbiosis of a gut microbiome is a key pathogenic mechanism in IBS. On day 7, the alpha diversity of the IBS and MIX groups was markedly lower than that of the NC group (Figure 5A, *p* < 0.05 for the IBS group, *p* < 0.001 for the MIX group), with their beta diversity also being substantially different from that of the NC group n(S4, *p* = 0.001 for the IBS group, *p* = 0.001 for the MIX group). By day 21, after the intervention with a complex probiotic, the α-diversity of the MIX group was markedly higher than that of the IBS group (Figure 5B, *p* < 0.0001), while the β-diversity revealed considerable disparities among the three groups (S5, *p* = 0.001 for each comparison). These results suggest that IBS is associated with reduced diversity and altered gut microbiota structure, while complex probiotic treatment can increase microbial diversity and restore gut microbiome composition.

**Figure 6:**
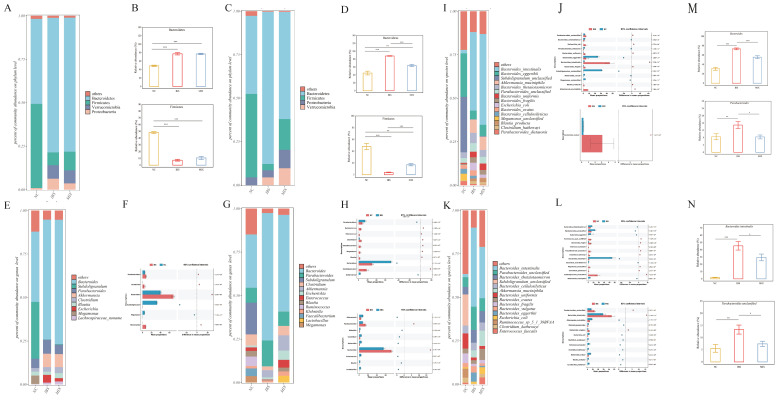
Treatment with a complex probiotic improved the gut microbiota in IBS mice (*n* = 10). (**A**) Gut microbiota composition at the phylum level on day 7. (**B**) Relative abundance of *Bacteroidetes* and *Firmicutes* on day 7. (**C**) Gut microbiota composition at the phylum level on day 21. (**D**) Relative abundance of *Bacteroidetes* and *Firmicutes* on day 21. (**E**) Gut microbiota composition of each group at the genus level on day 7. (**F**) Differential genera between the NC and IBS groups on day 7. (**G**) Gut microbiota composition of each group at the genus level on day 21. (**H**) Differential genera between the NC and IBS groups and between the IBS and MIX groups on day 21. (**I**) Gut microbiota composition at the species level on day 7. (**J**) Differential species between the NC and IBS groups as well as between the IBS and MIX groups on day 7. (**K**) Gut microbiota composition of each group at the species level on day 21. (**L**) Differential species between the NC and IBS groups as well as between the IBS and MIX groups on day 21. (**M**) Relative abundance of *Bacteroides* and *Parabacteroides* on day 21. (**N**) Relative abundance of *Bacteroides intestinalis* and *Parabacteroides unclassified* on day 21. Mann–Whitney rank sum test followed by Wilcoxon test. Notable disparities are signified by * (*p* < 0.05), ** (*p* < 0.01), *** (*p* < 0.001).

This research analyzed the microbial community structure of each fecal sample. On day 7, the dominant phyla were *Bacteroidetes*, *Verrucomicrobia* and *Firmicutes* (Figure 6A). Specifically, the IBS and MIX groups exhibited markedly elevated levels of *Bacteroidetes* but markedly lower levels of *Firmicutes* compared with the NC group (Figure 6B, *p* < 0.001 for Bacteroidetes, *p* < 0.001 for Firmicutes). However, by day 21, after the complex probiotic intervention, *Bacteroidetes* levels were markedly decreased (*p* < 0.001), while those of *Firmicutes* were markedly increased in the MIX group relative to the IBS group (Figure 6C,D, *p* < 0.001 for *Bacteroidetes*, *p* < 0.001 for *Firmicutes*).

STAMP analysis was used to identify differential genera in the gut microbiota of mice. On day 7, fecal transplantation from IBS patients resulted in a significant rise in the relative abundance of *Parabacteroides*, *Escherichia* and *Bacteroides,* along with a significant reduction in the relative abundance of *Subdoligranulum*, *Megamonas* and other genera. However, no differential genera were observed between the IBS and MIX groups at this stage (Figure 6E,F). By day 21, fecal transplantation from IBS patients continued to significantly increase the relative abundance of *Parabacteroides*, *Bacteroides* and other genera, while significantly reducing the relative abundance of *Subdoligranulum*, *Megamonas* and others. However, after intervention with a complex probiotic, the levels of *Parabacteroides* and *Bacteroides* were markedly decreased (*p* < 0.001), while those of *Akkermansia*, *Klebsiella* and *Lactobacillus* were markedly increased in the MIX group relative to the IBS group (Figure 6G,H). In particular, the rise in the relative abundance of *Parabacteroides* and *Bacteroides*, observed in the IBS group following fecal transplantation, was significantly reversed in the MIX group after the complex probiotic intervention (Figure 6M, *p* < 0.05 for *Parabacteroides*, *p* < 0.001 for *Bacteroides*).

At the species level, on day 7, fecal gavage from IBS patients led to a significant increase in the relative abundance of *Parabacteroides unclassified*, *Bacteroides intestinalis* and *Blautia producta,* as well as a significant decrease in the relative abundance of *Parabacteroides distasonis*, *Subdoligranulum unclassified* and *Bacteroides caccae*. In this case, one strain, *Bacteroides ovatus*, was differentially abundant between the IBS and MIX groups and it was excluded from subsequent analyses (Figure 6I,J). By day 21, fecal gavage from IBS patients continued to significantly increase the relative abundance of *Bacteroides intestinalis*, *Parabacteroides unclassified* and *Clostridium clostridioforme* while significantly decreasing the relative abundance of *Clostridium hathewayi*, *Megamonas unclassified* and *Subdoligranulum unclassified.* However, after the intervention with a complex probiotic, the MIX group exhibited significantly reduced levels of *Bacteroides intestinalis* and *Parabacteroides unclassified*, while *Klebsiella pneumoniae*, *Clostridium symbiosum*, *Clostridium hathewayi* and *Blautia producta* were significantly increased (Figure 6K,L). More importantly, the increase in the relative abundance of *Bacteroides intestinalis* and *Parabacteroides unclassified*, observed in the IBS group after fecal gavage, was significantly reversed in the MIX group after the complex probiotic intervention. Overall, the MIX group could successfully mitigate the increasing trend of these strains relative to the IBS group (Figure 6N, *p* < 0.05 for *Bacteroides intestinalis*, *p* < 0.05 for *Parabacteroides unclassified*).

### 3.5. Effects of the Complex Probiotic on Fecal Metabolites in IBS

**Figure 7:**
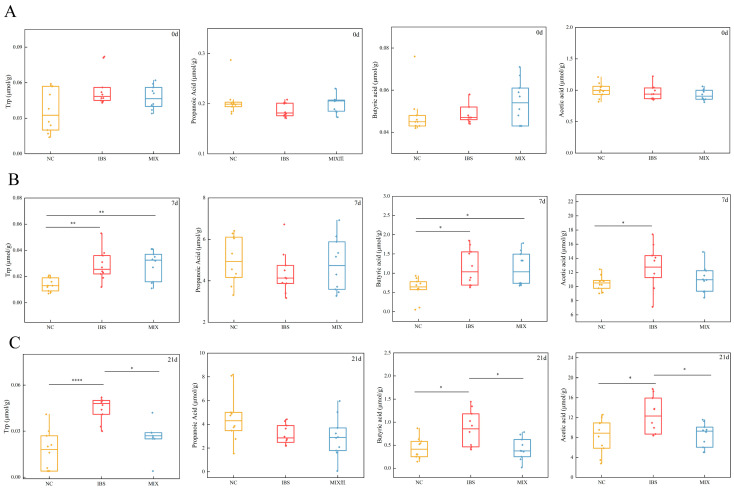
Treatment with the a complex probiotic improved the amount of short-chain fatty acids and tryptophan in the feces of IBS mice (*n* = 10). (**A**) Fecal levels of tryptophan, propionic acid, butyric acid and acetic acid on day 0. (**B**) Levels of tryptophan, propionic acid, butyric acid and acetic acid in feces on day 7. (**C**) Levels of tryptophan, propionic acid, butyric acid, and acetic acid in feces on day 21. Statistical analysis was performed using the Mann–Whitney test. Notable disparities are signified by * (*p* < 0.05), ** (*p* < 0.01), and **** (*p* < 0.0001).

Gut microbiota play a crucial role in the metabolism of intestinal components by producing metabolites that may directly or indirectly contribute to IBS symptoms. To investigate the metabolic changes in IBS and the effects of a complex probiotic on these metabolites, this study analyzed fecal samples using targeted metabolomics. The results, shown in Figure 7, indicate that on day 0, no noteworthy disparities were observed in any of the metabolites across the groups (Figure 7A). By day 7, levels of tryptophan, butyric acid and acetic acid were significantly higher in the IBS and/or MIX groups (both received fecal microbiota from IBS patients) compared with the NC group (received healthy human fecal microbiota) (Figure 7B, *p* < 0.01, *p* < 0.05, *p* < 0.05). On day 21, following the intervention with a complex probiotic, the levels of tryptophan, butyric acid and acetic acid were markedly lower in the MIX group relative to the IBS group (Figure 7C, *p* < 0.05, *p* < 0.05, *p* < 0.05, *p* < 0.05), with these values being close to those of the normal group. However, propionic acid levels were not significantly different on day 7 or day 21.

### 3.6. Correlation Analysis Between Gut Microbiota and Environmental Factors

**Figure 8:**
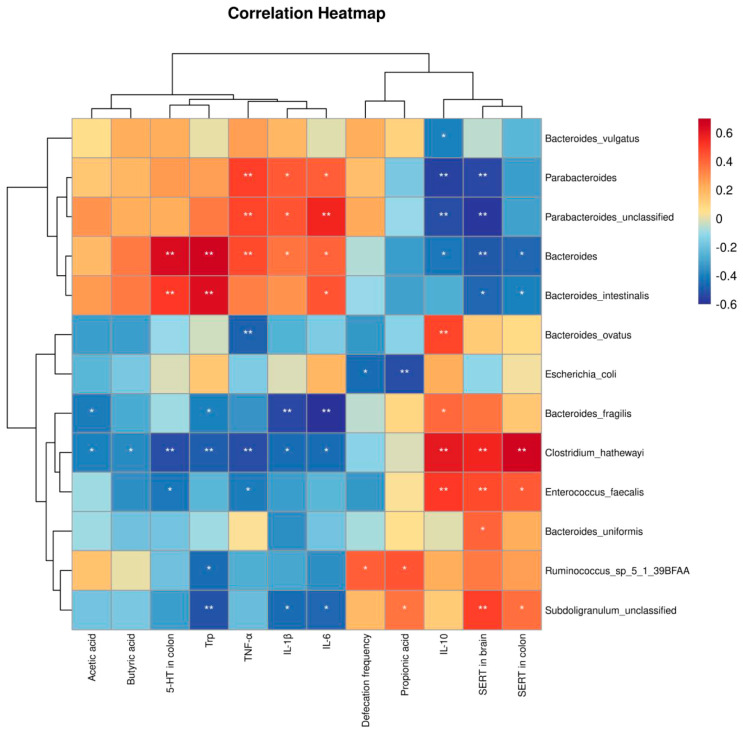
The Spearman’s rank correlation coefficient was utilized to evaluate the association between the gut microbiome and environmental elements. Red and blue hues represent positive and negative correlations, respectively, with the color saturation reflecting the correlation’s magnitude.Notable disparities are signified by * (*p* < 0.05), ** (*p* < 0.01).

To further investigate whether a complex probiotic can alleviate IBS symptoms through its effects on gut microbiota and metabolites, Pearson correlation analysis was performed (Figure 8). The data indicated that *Bacteroides intestinalis* had a significant positive correlation with tryptophan, IL-6 and 5-HT in the colon (R = 0.626, R = 0.444, R = 0.506) and a significantly negative correlation with SERT in the colon and brain tissues (R = −0.393, R = −0.461). Furthermore, *Bacteroides intestinalis* showed a positive but not significant correlation with acetic acid and butyric acid (R = 0.277, R = 0.346). On the other hand, *Parabacteroides unclassified* exhibited a significant negative correlation with IL-10 and SERT in brain tissues (R = −0.530, R = −0.575) and a significant positive correlation with TNF-α, IL-1β and IL-6 (R = 0.481, R = 0.442, R = 0.567).

## 4. Discussion

The pathogenesis of IBS is multifactorial and remains unclear. However, growing research implies that alterations in gut microbiota may contribute to the development and persistence of this condition [34]. Currently, there is no ideal treatment for IBS, but probiotics, as beneficial microorganisms, have shown promise in regulating the gut microbiota [35]. Indeed, the intake of complex probiotics has been shown to reduce the severity of IBS symptoms [36]; hence, the aim of this research was to explore the relationship between gut microbiota and phenotypic indicators of IBS following intervention with complex probiotic. The results indicated that complex probiotics could alleviate IBS symptoms in mice by reducing serum inflammatory factor levels, modulating gut microbiota and altering fecal metabolites.

The main symptoms of IBS include abdominal pain and abnormal bowel movements characterized by changes in bowel frequency and/or stool consistency. Diarrhea is a major symptom of IBS, and the number of fecal pellets and water content in a 2-h bowel movement are key indicators of diarrhea severity. This study found that, by day 7, both the number of fecal pellets and fecal water content were markedly elevated in the IBS and MIX groups relative to the NC group. However, by day 21, the number of fecal pellets was significantly reduced in the MIX group compared with the IBS group, thus indicating that, even though the animals developed diarrhea symptoms after modeling, these symptoms could be partially alleviated by the intervention of a complex probiotic. While there is a relationship between intake and the number of bowel movements, the underlying factor lies in the food composition; for example, diets low in FODMAPs (fermentable carbohydrates reduced) have been shown to improve symptoms of irritable bowel syndrome, whereas high-fat or high-fiber diets may affect bowel patterns by altering intestinal osmolality or fermentative gas production [37]. No specific FODMAP ingredients were added to the diets in this study, and all mice were on a standardized diet; however, our study focused on changes in fecal pellet counts rather than directly exploring the effects of dietary intake on symptoms. Therefore, we did not strictly measure food intake in our experiments. Although changes in intestinal flora and metabolism occurred in this study, these changes were not always directly reflected in histologic scores. For example, in some animal models of irritable bowel syndrome, abnormalities in intestinal tissue structure may not be apparent despite significant changes in intestinal flora and metabolism [38]. Second, histologic scoring focuses on changes in intestinal tissue structure, such as mucosal inflammation, cellular proliferation, or intestinal wall thickness. However, the pathology of irritable bowel syndrome is characterized more by dysfunction than by structural damage [1]. Therefore, even if there are significant changes in intestinal flora and metabolism, these changes may not cause significant alterations in histologic scores. Furthermore, previous reports have shown that multi-strain probiotics can effectively relieve IBS-related symptoms. For instance, Abdellah et al. [39] demonstrated that multi-strain probiotics may reduce intestinal permeability, improve diarrhea, enhance stool consistency and boost life satisfaction for a subset of patients. Similarly, a 28-day multicenter, randomized, double-blind and placebo-controlled study by Friedman et al. [40] found that administering multiple probiotic strains significantly reduced episodes of diarrhea in IBS-D patients relative to a placebo group.

Low-grade mucosal inflammation and immune activation might underlie IBS pathogenesis and could also function as potential diagnostic markers for the condition. Furthermore, high levels of pro-inflammatory cytokines tend to be associated with the anxiety and depression often seen in IBS patients. Therefore, reducing serum inflammatory factor levels may represent a key strategy for treating this condition. In this study, the levels of serum pro-inflammatory factors TNF-α, IL-6 and IL-1β were markedly higher, while those of the serum anti-inflammatory factor IL-10 were markedly lower in the IBS group relative to the NC group. However, intervention with a complex probiotic significantly lowered the serum pro-inflammatory factors TNF-α, IL-6 and IL-1β while markedly increasing the serum anti-inflammatory factor IL-10 in the MIX group relative to the IBS group. These results suggest that the complex probiotic can effectively reduce serum inflammatory factor levels in IBS mice while also possibly alleviating clinical symptoms such as diarrhea, abdominal pain, anxiety and depression. These findings were also consistent with those of Wang et al. [41], who induced a PI-IBS model using spinosyn infection. After intervention with three probiotic mixtures, it was found that the IL-6 levels were markedly reduced in the probiotic group relative to the IBS group. Furthermore, Brun et al. [42] found that *Saccharomyces boulardii CNCM I-745* treatment markedly decreased the levels of pro-inflammatory cytokines TNF-α and IL-1β while increasing the levels of the anti-inflammatory factor IL-10, thereby reducing serum inflammatory factor levels in mice with herpes simplex virus type 1.

Dysbiosis of the gut microbiota is widely considered one of the vital pathogenic mechanisms that lead to IBS, and therefore, correcting gut microbiota imbalances and restoring the levels of various bacterial genera is crucial for the treatment of IBS [43]. In this study, fecal samples were collected to analyze the composition of the intestinal microbiota. Typically, the gut microbiota is dominated by *Firmicutes* and *Bacteroidetes* [16]. However, due to individual differences, research findings tend to vary: Some studies report a decrease in *Firmicutes*, a rise in *Bacteroidetes* and a decrease in the *Firmicutes*/*Bacteroidetes* ratio in IBS patients, while other studies report the exact opposite [44,45]. It has since been suggested that patients with different subtypes of IBS have different *Firmicutes*/*Bacteroidetes* ratios, with patients with IBS-D usually having decreased *Firmicutes*/*Bacteroidetes* and patients with IBS-C having increased *Firmicutes*/*Bacteroidetes* [46]. In this study, by day 7, there was a marked rise in *Bacteroidetes* and a marked decline in *Firmicutes* in both the IBS and MIX groups relative to the NC group at the phylum level. This is consistent with the previous point that the *Firmicutes*/*Bacteroidetes* ratio is decreasing.

The gut microbiota of the mice was then further analyzed at the genus level, with the results indicating that the relative abundance of *Parabacteroides* and *Bacteroidetes* was markedly higher in the IBS and MIX groups relative to the NC group. However, the intervention with a complex probiotic markedly decreased the levels of *Parabacteroides* and *Bacteroidetes* in the MIX group relative to the IBS group. It has been reported that a higher abundance of *Bacteroidetes* is associated with intestinal inflammation as its representative strains tend to be pro-inflammatory [47]. In addition, another study also noted that the abundance of *Bacteroidetes* was higher in IBS-D patients than in healthy controls, which may be related to the metabolic function and inflammatory response of the intestinal mucosa [48]. Similarly, *Parabacteroides* is recognized as a potential pathogen [49]. The IBS flora is characterized by a decrease in the number of probiotic bacteria and an increase in the number of pathogenic bacteria. One study suggests that an increase in *Parabacteroides* may contribute to impaired intestinal barrier function and an increased inflammatory response [46]. In this research, the results of correlation analysis further showed a significant negative correlation between *Bacteroidetes and Parabacteroides* and serum anti-inflammatory factor IL-10 as well as a significant positive correlation with serum pro-inflammatory factors IL-1β, IL-6 and TNF-α; these findings align with those of previous research. The relative content of *Bacteroidetes and Parabacteroides* can be reduced after complex probiotic intervention, which, in turn, reduces serum inflammatory factor levels. Finally, the analysis was performed at the species level, and the results showed that the relative abundance of *Bacteroides intestinalis* and *Parabacteroides unclassified* in mice that received fecal microbiota from IBS patients was significantly higher than that of mice that received fecal microbiota from healthy individuals on day 7. However, supplementation with a complex probiotic significantly reduced the relative abundance of *Bacteroides intestinalis* and *Parabacteroides unclassified* in the MIX group compared with the IBS group. *Bacteroides intestinalis* belongs to the *Bacteroidetes* phylum, whose members are known to degrade complex arabinoxylans and xylans in dietary fibers, such as those found in wheat, rye, oats and barley. These degradation products, which include butyrate and ferulic acid, have been shown to protect the intestinal mucosa [50]. Furthermore, research has shown that the complex arabinoxylan products degraded by *Bacteroides intestinalis* could exert anti-inflammatory activity on dendritic cells under inflammatory conditions and enhance Th1-type immune responses in mice under physiological conditions [51]. Costa et al. [52] also investigated whether there was an association between the inflammatory potential of the diet and gut microbiota composition in adults with functional constipation. In this case, 68 patients with functional constipation were divided into two groups based on their dietary patterns—anti-inflammatory and pro-inflammatory—classified according to the Dietary Inflammatory Index. It was found that the relative abundance of *Bacteroides intestinalis* was markedly lower in the anti-inflammatory group. Previous research has shown that a higher relative abundance of *Bacteroides intestinalis* was linked to inflammatory factor levels, which aligns with the findings of the present research. Subsequent correlation analyses further showed that *Bacteroides intestinalis* was significantly and positively correlated with IL-6. In contrast, *Parabacteroides unclassified* is yet to be classified and therefore, it lacks extensive literature support. Nevertheless, previous research suggests that *Parabacteroides* was linked to inflammation. Furthermore, a correlation study revealed a markedly inverse relationship between *Parabacteroides unclassified* and IL-10 but a markedly direct relationship with IL-1β, IL-6 and TNF-α. Hence, the results indicated that *Parabacteroides unclassified* can have potential anti-inflammatory effects. In conclusion, complex probiotics appear to restore gut microbiota by reducing the relative abundance of *Bacteroides intestinalis* and *Parabacteroides unclassified*, thereby reducing the levels of serum inflammatory factors.

5-Hydroxytryptamine (5-HT), also known as serotonin, is a key neurotransmitter and signaling molecule in the brain–gut axis [53]. Approximately 90–95% of 5-HT is produced in the gut, and 5-HT exerts its biological effects by binding to a variety of 5-HT receptors, of which 5-HT3 and 5-HT4 have been implicated in the pathogenesis of IBS [54]. The reuptake of 5-HT takes place mainly through serotonin transporter (SERT), which transports 5-HT into cells, where 5-HT is degraded and metabolites are eliminated from the body via the kidneys [55]. Thus, SERT expression controls local 5-HT levels to some extent, the increase in 5-HT is associated with accelerated intestinal transit and enhanced luminal endocytosis [56]. Dysregulation of 5-HT metabolism is involved in the pathogenesis of several diseases characterized by intestinal motility disorders [57]. In IBS-D or diarrheal disease, reductions in SERT or serial enhancement of 5-HT have been reported more frequently in previous studies [58,59]. For example, Meng et al. [60] Found that transplanted cecal matter from WAS group rats into normal rats increased the level of 5-HT and decreased the level of SERT in the colon. Meanwhile, a decrease in SERT expression may lead to an increase in 5-HT levels. The results of this study showed that in the colon tissue, the 5-HT content of IBS group mice was significantly increased, and the SERT content was significantly decreased; in brain tissue, the 5-HT content of IBS group mice tended to increase but did not show a significant difference, while the SERT content was significantly decreased. After the intervention with a probiotic complex, the 5-HT content was significantly reduced, and SERT content was elevated in the colon tissue. In brain tissue, there was a tendency to reduce 5-HT, but no significant difference was observed, while SERT was significantly increased. It is well known that intestinal 5-HT is positively correlated with intestinal motility. We consistently found that two-hourly defecation pellet counts were significantly reduced, and intestinal motility was inhibited in mice after probiotic complex intervention. These results suggest that probiotic complexes can inhibit intestinal motility by upregulating SERT expression and reducing 5-HT levels.

Short-chain fatty acids (SCFAs), such as acetic acid, propionic acid and butyric acid, are not only the main by-products of intestinal microbial metabolism [61] but also the most abundant SCFAs in the colon, accounting for 90–95% of the SCFAs [62]. Butyric acid, in particular, plays a crucial role by activating the hydroxycarboxylic acid receptor HCAR2 (GPR109A) in colonic macrophages and dendritic cells. This activation induces the differentiation of regulatory T-cells and promotes the production of the anti-inflammatory cytokine IL-10, thereby reducing intestinal inflammation [63]. However, in the present study, butyric acid was found to be elevated in the IBS group and decreased in the MIX group, which may be due to the fact that butyric acid needs to reach a certain threshold concentration to exert its anti-inflammatory effects [64]. Below this threshold [65,66], the anti-inflammatory effect of butyric acid may be insignificant or completely ineffective, and it has also been reported that butyric acid can reduce inflammation by promoting the production of IL-10 in certain inflammatory conditions. In this experiment, although butyric acid levels were elevated in the IBS group, the low levels may not have reached the threshold for anti-inflammatory effects, or the low levels of butyric acid may not have produced enough IL-10 to have an effect on the overall results. SCFAs are also key regulators of intestinal motility [67]. Tryptophan, the main precursor of serotonin, is another important metabolite, with both a deficiency and an excess of the latter possibly leading to gastrointestinal dysfunction [68]. In this context, a study found that reducing the dietary intake of tryptophan could reduce abdominal symptoms without interfering with the mental status of patients with diarrhea-predominant IBS [69]. In this experiment, the levels of tryptophan, butyric acid, acetic acid and propionic acid in mouse feces were determined by targeted metabolomics. The results showed that the levels of tryptophan, butyric acid, and acetic acid were markedly elevated at the end of modeling. However, on day 21, after the intervention with the complex probiotic, these levels were markedly reduced in the MIX group relative to the IBS group. These outcomes imply that the complex probiotic can improve the abnormal fecal metabolite levels in IBS mice. Additionally, a randomized, double-blind, placebo-controlled, multicenter trial found that probiotics significantly increased the fecal levels of acetic acid and butyric acid [70]. In addition, Zhou et al. [71] observed that the levels of propionic acid, butyric acid, acetic acid and tryptophan were lower in the IBS group relative to the NC group. However, the levels of these metabolites showed a rising trend in the IBS + Bifico group, which was supplemented with the complex probiotic compared with the IBS group. A different study further reported significantly higher levels of tryptophan in the feces of IBS rats [72], along with a significant increase in 5-HT metabolites. These levels subsequently returned to normal after intragastric administration of *Lactobacillus plantarum AR495*. Altogether, the findings are consistent with previous ones where 5-HT levels in the colon of IBS mice were significantly reduced after the complex probiotic intervention. Finally, correlation analysis revealed that tryptophan was significantly and positively correlated with *Bacteroides*. A separate study [73] involving a prolonged microgravity exposure in mice suggested that this particular condition increased susceptibility to depression, with *Bacteroides* showing a positive correlation with tryptophan metabolism.

## 5. Conclusions

In summary, this study demonstrated that the complex probiotic significantly reduced the number of 2-h defecation pellets and regulated intestinal motility by normalizing abnormal 5-HT and SERT levels, thereby reducing diarrhea symptoms in IBS. In addition, the complex probiotic effectively reduced serum inflammatory factor levels by decreasing serum pro-inflammatory factors and increasing serum anti-inflammatory ones. It also restored gut microbiota diversity and structure by modulating the IBS-associated microbiota and reducing the relative abundance of inflammation-associated strains such as *Bacteroides intestinalis* and *Parabacteroides unclassified*. Finally, the complex probiotic helped to balance intestinal metabolism by restoring fecal levels of butyric acid, acetic acid and tryptophan. However, further research using transcriptomics and proteomics, as well as larger clinical studies, would be required to gain deeper insights into the molecular mechanisms of the complex probiotic and to validate its feasibility and effectiveness in clinical practice. The enterovirus group was not considered in this study, but future studies will examine the effect of the enterovirus group on irritable bowel syndrome.

## Data Availability

The original contributions presented in the study are included in the article/Supplementary Materials, further inquiries can be directed to the corresponding authors.

## Supplementary Materials

The following supporting information can be downloaded at: https://www.mdpi.com/article/10.3390/nu17050801/s1, Table S1: IBS Donor Patient Information; Table S2: HE staining scoring criteria for enterocolitis; Table S3: The LC (liquid chromatography) programmed elution gradient for determination of SCFAs (short-chain fatty acids); Table S4: Analysis of β diversity differences between groups on day 7; Table S5: Analysis of β diversity difference between groups on day 21.

## References

[1] Chey W.D., Kurlander J., Eswaran S. (2015). Irritable bowel syndrome: A clinical review. JAMA.

[2] Mearin F., Lacy B.E., Chang L., Chey W.D., Lembo A.J., Simren M., Spiller R. (2016). Bowel disorders. Gastroenterology.

[3] Palsson O.S., Whitehead W., Törnblom H., Sperber A.D., Simren M. (2020). Prevalence of Rome IV functional bowel disorders among adults in the United States, Canada, and the United Kingdom. Gastroenterology.

[4] Spiller R., Major G. (2016). IBS and IBD—Separate entities or on a spectrum?. Nat. Rev. Gastroenterol. Hepatol..

[5] Vasant D.H., A Paine P., Black C.J., A Houghton L., Everitt H.A., Corsetti M., Agrawal A., Aziz I., Farmer A.D., Eugenicos M.P. (2022). British Society of Gastroenterology Guidelines for the Management of Irritable Bowel Syndrome. Gut.

[6] Distrutti E., Monaldi L., Ricci P., Fiorucci S. (2016). Gut microbiota role in irritable bowel syndrome: New therapeutic strategies. World J. Gastroenterol..

[7] Napolitano M., Fasulo E., Ungaro F., Massimino L., Sinagra E., Danese S., Mandarino F.V. (2023). Gut dysbiosis in irritable bowel syndrome: A narrative review on correlation with disease subtypes and novel therapeutic implications. Microorganisms.

[8] Eswaran S., Chey W.D., Jackson K., Pillai S., Chey S.W., Han-Markey T. (2017). A diet low in fermentable oligo-, di-, and monosaccharides and polyols improves quality of life and reduces activity impairment in patients with irritable bowel syndrome and diarrhea. Clin. Gastroenterol. Hepatol..

[9] Zheng T., Eswaran S., Photenhauer A.L., Merchant J.L., Chey W.D., D’amato M. (2020). Reduced efficacy of low FODMAPs diet in patients with IBS-D carrying sucrase-isomaltase (*SI*) hypomorphic variants. Gut.

[10] Staudacher H.M., Whelan K. (2017). The low FODMAP diet: Recent advances in understanding its mechanisms and efficacy in IBS. Gut.

[11] Camilleri M., Boeckxstaens G. (2017). Dietary and pharmacological treatment of abdominal pain in IBS. Gut.

[12] Lacy B.E., Patel N.K. (2017). Rome criteria and a diagnostic approach to irritable bowel syndrome. J. Clin. Med..

[13] Hill C., Guarner F., Reid G., Gibson G.R., Merenstein D.J., Pot B., Morelli L., Canani R.B., Flint H.J., Salminen S. (2014). Expert consensus document: The International Scientific Association for Probiotics and Prebiotics consensus statement on the scope and appropriate use of the term probiotic. Nat. Rev. Gastroenterol. Hepatol..

[14] Collado M.C., Gueimonde M., Sanz Y., Salminen S. (2006). Adhesion properties and competitive pathogen exclusion ability of bifidobacteria with acquired acid resistance. J. Food Prot..

[15] Vieira A.T., Teixeira M.M., Martins F.S. (2013). The role of probiotics and prebiotics in inducing gut immunity. Front. Immunol..

[16] Zhuang X., Tian Z., Li L., Zeng Z., Chen M., Xiong L. (2018). Fecal microbiota alterations associated with diarrhea-predominant irritable bowel syndrome. Front. Microbiol..

[17] Jeffery I.B., O’Toole P.W., Öhman L., Claesson M.J., Deane J., Quigley E.M.M., Simrén M. (2011). An irritable bowel syndrome subtype defined by species-specific alterations in faecal microbiota. Gut.

[18] Sun Y.-Y., Li M., Li Y.-Y., Li L.-X., Zhai W.-Z., Wang P., Yang X.-X., Gu X., Song L.-J., Li Z. (2018). The effect of *Clostridium* butyricum on symptoms and fecal microbiota in diarrhea-dominant irritable bowel syndrome: A randomized, double-blind, placebo-controlled trial. Sci. Rep..

[19] Niu H.-L., Xiao J.-Y. (2020). The efficacy and safety of probiotics in patients with irritable bowel syndrome: Evidence based on 35 randomized controlled trials. Int. J. Surg..

[20] Preston K., Krumian R., Hattner J., de Montigny D., Stewart M., Gaddam S. (2018). Lactobacillus acidophilus CL1285, Lactobacillus casei LBC80R and Lactobacillus rhamnosus CLR2 improve quality-of-life and IBS symptoms: A double-blind, randomised, placebo-controlled study. Benef. Microbes.

[21] Wang Y., Li Y., Xie J., Zhang Y., Wang J., Sun X., Zhang H. (2013). Protective effects of probiotic Lactobacillus casei Zhang against endotoxin- and d-galactosamine-induced liver injury in rats via anti-oxidative and anti-inflammatory capacities. Int. Immunopharmacol..

[22] Wang L., Liu C., Chen M., Ya T., Huang W., Gao P., Zhang H. (2015). A novel Lactobacillus plantarum strain P-8 activates beneficial immune response of broiler chickens. Int. Immunopharmacol..

[23] Wang J., Bai X., Peng C., Yu Z., Li B., Zhang W., Sun Z., Zhang H. (2020). Fermented milk containing Lactobacillus casei Zhang and Bifidobacterium animalis ssp. lactis V9 alleviated constipation symptoms through regulation of intestinal microbiota, inflammation, and metabolic pathways. J. Dairy Sci..

[24] Kwok L., Wang L., Zhang J., Guo Z., Zhang H. (2014). A pilot study on the effect of Lactobacillus casei Zhang on intestinal microbiota parameters in Chinese subjects of different age. Benef. Microbes.

[25] Wang L., Zhang J., Guo Z., Kwok L., Ma C., Zhang W., Lv Q., Huang W., Zhang H. (2014). Effect of oral consumption of probiotic Lactobacillus planatarum P-8 on fecal microbiota, SIgA, SCFAs, and TBAs of adults of different ages. Nutrition.

[26] Xu H., Ma C., Zhao F., Chen P., Liu Y., Sun Z., Cui L., Kwok L.-Y., Zhang H. (2021). Adjunctive treatment with probiotics partially alleviates symptoms and reduces inflammation in patients with irritable bowel syndrome. Eur. J. Nutr..

[27] Tawfeeq H.R., Lackey A.I., Zhou Y., Diolintzi A., Zacharisen S.M., Lau Y.H., Quadro L., Storch J. (2025). Tissue-Specific Ablation of Liver Fatty Acid-Binding Protein Induces a Metabolically Healthy Obese Phenotype in Female Mice. Nutrients.

[28] Tan S., Gu J., Yang J., Dang X., Liu K., Gong Z., Xiao W.J. (2025). L-Theanine Mitigates Acute Alcoholic Intestinal Injury by Activating the HIF-1 Signaling Pathway to Regulate the TLR4/NF-κB/HIF-1α Axis in Mice. Nutrients.

[29] Cai B., Zhou M.-H., Huang H.-L., Zhou A.-C., Chu Z.-D., Huang X.-D., Li C.-W. (2020). Protective effects of citrulline supplementation in ulcerative colitis rats. PLoS ONE.

[30] Zhao Y., Zhan J., Sun C., Zhu S., Zhai Y., Dai Y., Wang X., Gao X. (2024). Sishen Wan enhances intestinal barrier function via regulating endoplasmic reticulum stress to improve mice with diarrheal irritable bowel syndrome. Phytomedicine.

[31] Yang J., Li H., Hao Z., Jing X., Zhao Y., Cheng X., Ma H., Wang J., Wang J. (2022). Mitigation effects of selenium nanoparticles on depression-like behavior induced by fluoride in mice via the JAK2-STAT3 pathway. ACS Appl. Mater. Interfaces.

[32] He D.-F., Ren Y.-P., Liu M.-Y. (2016). Effects of ginseng fruit saponins on serotonin system in Sprague-Dawley rats with myocardial infarction, depression, and myocardial infarction complicated with depression. Chin. Med. J..

[33] Li B., Hui F., Yuan Z., Shang Q., Shuai G., Bao Y., Chen Y. (2021). Untargeted fecal metabolomics revealed biochemical mechanisms of the blood lipid-lowering effect of koumiss treatment in patients with hyperlipidemia. J. Funct. Foods.

[34] Pozuelo M., Panda S., Santiago A., Mendez S., Accarino A., Santos J., Guarner F., Azpiroz F., Manichanh C. (2015). Reduction of butyrate- and methane-producing microorganisms in patients with Irritable Bowel Syndrome. Sci. Rep..

[35] Stavrou G. (2016). Gut microbiome, surgical complications and probiotics. Ann. Gastroenterol..

[36] Skrzydło-Radomańska B., Prozorow-Król B., Cichoż-Lach H., Majsiak E., Bierła J.B., Kanarek E., Sowińska A., Cukrowska B. (2021). The effectiveness and safety of multi-strain probiotic preparation in patients with diarrhea-predominant irritable bowel syndrome: A randomized controlled study. Nutrients.

[37] Eswaran S.L., Chey W.D., Han-Markey T., Ball S., Jackson K. (2016). A Randomized Controlled Trial Comparing the Low FODMAP Diet vs. Modified NICE Guidelines in US Adults with IBS-D. Am. J. Gastroenterol..

[38] Orlando A., Chimienti G., Notarnicola M., Russo F. (2022). The ketogenic diet improves gut–brain axis in a rat model of irritable bowel syndrome: Impact on 5-HT and BDNF systems. Int. J. Mol. Sci..

[39] Abdellah S.A., Gal C., Laterza L., Velenza V., Settanni C.R., Napoli M., Schiavoni E., Mora V., Petito V., Gasbarrini A. (2022). Effect of a multistrain probiotic on leaky gut in patients with diarrhea-predominant irritable bowel syndrome: A pilot study. Dig. Dis..

[40] Friedman G. (2008). A Multi-Strain Probiotic Reduces the Frequency of Diarrhea in IBS-D Patients. Am. J. Gastroenterol..

[41] Wang H., Gong J., Wang W., Long Y., Fu X., Fu Y., Qian W., Hou X. (2014). Are there any different effects of Bifidobacterium, Lactobacillus and Streptococcus on intestinal sensation, barrier function and intestinal immunity in PI-IBS mouse model?. PLoS ONE.

[42] Brun P., Scarpa M., Marchiori C., Sarasin G., Caputi V., Porzionato A., Giron M.C., Palù G., Castagliuolo I. (2017). Saccharomyces boulardii CNCM I-745 supplementation reduces gastrointestinal dysfunction in an animal model of IBS. PLoS ONE.

[43] Si J.-M., Yu Y.-C., Fan Y.-J., Chen S.-J. (2004). Intestinal microecology and quality of life in irritable bowel syndrome patients. World J. Gastroenterol..

[44] Rajilić–Stojanović M., Biagi E., Heilig H.G., Kajander K., Kekkonen R.A., Tims S., de Vos W.M. (2011). Global and deep molecular analysis of microbiota signatures in fecal samples from patients with irritable bowel syndrome. Gastroenterology.

[45] Jalanka-Tuovinen J., Salojärvi J., Salonen A., Immonen O., Garsed K., Kelly F.M., Zaitoun A., Palva A., Spiller R.C., de Vos W.M. (2013). Faecal microbiota composition and host–microbe cross-talk following gastroenteritis and in postinfectious irritable bowel syndrome. Gut.

[46] Gryaznova M., Smirnova Y., Burakova I., Morozova P., Lagutina S., Chizhkov P., Korneeva O., Syromyatnikov M. (2024). Fecal Microbiota Characteristics in Constipation-Predominant and Mixed-Type Irritable Bowel Syndrome. Microorganisms.

[47] Ryan F.J., Ahern A.M., Fitzgerald R.S., Laserna-Mendieta E.J., Power E.M., Clooney A.G., O’donoghue K.W., McMurdie P.J., Iwai S., Crits-Christoph A. (2020). Colonic microbiota is associated with inflammation and host epigenomic alterations in inflammatory bowel disease. Nat. Commun..

[48] Olyaiee A., Sadeghi A., Yadegar A., Mirsamadi E.S., Mirjalali H. (2022). Gut Microbiota Shifting in Irritable Bowel Syndrome: The Mysterious Role of *Blastocystis* sp.. Front. Med..

[49] Yan Q., Gu Y., Li X., Yang W., Jia L., Chen C., Han X., Huang Y., Zhao L., Li P. (2017). Alterations of the gut microbiome in hypertension. Front. Cell. Infect. Microbiol..

[50] El Mouzan M., Assiri A., Al Sarkhy A. (2023). Gut microbiota predicts the diagnosis of celiac disease in Saudi children. World J. Gastroenterol..

[51] Yasuma T., Toda M., Abdel-Hamid A.M., D’alessandro-Gabazza C., Kobayashi T., Nishihama K., D’alessandro V.F., Pereira G.V., Mackie R.I., Gabazza E.C. (2021). Degradation products of complex arabinoxylans by Bacteroides intestinalis enhance the host immune response. Microorganisms.

[52] Costa L.M., Mendes M.M., Oliveira A.C., Magalhães K.G., Shivappa N., Hebert J.R., da Costa T.H.M., Botelho P.B. (2022). Dietary inflammatory index and its relationship with gut microbiota in individuals with intestinal constipation: A cross-sectional study. Eur. J. Nutr..

[53] Kim D.-Y., Camilleri M. (2000). Serotonin: A mediator of the brain–gut connection. Am. J. Gastroenterol..

[54] Vahora I.S., Tsouklidis N., Kumar R., Soni R., Khan S. (2020). How serotonin level fluctuation affects the effectiveness of treatment in irritable bowel syndrome. Cureus.

[55] Bertrand P.P., Bertrand R.L. (2010). Serotonin release and uptake in the gastrointestinal tract. Auton. Neurosci..

[56] Yano J.M., Yu K., Donaldson G.P., Shastri G.G., Ann P., Ma L., Nagler C.R., Ismagilov R.F., Mazmanian S.K., Hsiao E.Y. (2015). Indigenous bacteria from the gut microbiota regulate host serotonin biosynthesis. Cell.

[57] Mawe G.M., Hoffman J.M. (2013). Serotonin signalling in the gut—Functions, dysfunctions and therapeutic targets. Nat. Rev. Gastroenterol. Hepatol..

[58] Dong Y., Han Y., Wang Z., Qin Z., Yang C., Cao J., Chen Y. (2017). Role of serotonin on the intestinal mucosal immune response to stress-induced diarrhea in weaning mice. BMC Gastroenterol..

[59] Bearcroft C.P., Perrett D., Farthing M.J.G. (1998). Postprandial plasma 5-hydroxytryptamine in diarrhoea predominant irritable bowel syndrome: A pilot study. Gut.

[60] Meng Y., Feng Y., Hang L., Zhou Y., Wang E., Yuan J. (2022). No synergistic effect of fecal microbiota transplantation and shugan decoction in water avoidance stress-induced IBS-D rat model. Front. Microbiol..

[61] Jiang W., Wu J., Zhu S., Xin L., Yu C., Shen Z. (2022). The role of short chain fatty acids in irritable bowel syndrome. J. Neurogastroenterol. Motil..

[62] Ríos-Covián D., Ruas-Madiedo P., Margolles A., Gueimonde M., De Los Reyes-Gavilán C.G., Salazar N. (2016). Intestinal short chain fatty acids and their link with diet and human health. Front. Microbiol..

[63] Singh N., Gurav A., Sivaprakasam S., Brady E., Padia R., Shi H., Thangaraju M., Prasad P.D., Manicassamy S., Munn D.H. (2014). Activation of Gpr109a, receptor for niacin and the commensal metabolite butyrate, suppresses colonic inflammation and carcinogenesis. Immunity.

[64] Sun J., Chen S., Zang D., Sun H., Sun Y., Chen J. (2024). Butyrate as a promising therapeutic target in cancer: From pathogenesis to clinic. Int. J. Oncol..

[65] Lee C., Kim B.G., Kim J.H., Chun J., Im J.P., Kim J.S. (2017). Sodium butyrate inhibits the NF-kappa B signaling pathway and histone deacetylation, and attenuates experimental colitis in an IL-10 independent manner. Int. Immunopharmacol..

[66] Siddiqui M.T., Cresci G.A.M. (2021). The immunomodulatory functions of butyrate. J. Inflamm. Res..

[67] Shaidullov I.F., Sorokina D.M., Sitdikov F.G., Hermann A., Abdulkhakov S.R., Sitdikova G.F. (2021). Short chain fatty acids and colon motility in a mouse model of irritable bowel syndrome. BMC Gastroenterol..

[68] Gros M., Gros B., Mesonero J.E., Latorre E. (2021). Neurotransmitter dysfunction in irritable bowel syndrome: Emerging approaches for management. J. Clin. Med..

[69] Chojnacki C., Medrek-Socha M., Blonska A., Zajdel R., Chojnacki J., Poplawski T. (2022). A Reduced Tryptophan Diet in Patients with Diarrhoea-Predominant Irritable Bowel Syndrome Improves Their Abdominal Symptoms and Their Quality of Life through Reduction of Serotonin Levels and Its Urinary Metabolites. Int. J. Mol. Sci..

[70] Bai T., Xu Z., Xia P., Feng Y., Liu B., Liu H., Chen Y., Yan G., Lv B., Yan Z. (2022). The short-term efficacy of bifidobacterium quadruple viable tablet in patients with diarrhea-predominant irritable bowel syndrome: Potentially mediated by metabolism rather than diversity regulation. Am. J. Gastroenterol..

[71] Zhou Y., Zhang F., Mao L., Feng T., Wang K., Xu M., Lv B., Wang X. (2023). Bifico relieves irritable bowel syndrome by regulating gut microbiota dysbiosis and inflammatory cytokines. Eur. J. Nutr..

[72] Zhang H., Xia Y., Wang G., Xiong Z., Wei G., Liao Z., Qian Y., Cai Z., Ai L. (2024). *Lactobacillus plantarum* AR495 improves colonic transport hyperactivity in irritable bowel syndrome through tryptophan metabolism. Food Funct..

[73] Yuan L., Zhang R., Li X., Gao C., Hu X., Hussain S., Zhang L., Wang M., Ma X., Pan Q. (2023). Long-term simulated microgravity alters gut microbiota and metabolome in mice. Front. Microbiol..

